# Prompt engineering with a large language model to assist providers in responding to patient inquiries: a real-time implementation in the electronic health record

**DOI:** 10.1093/jamiaopen/ooae080

**Published:** 2024-08-20

**Authors:** Majid Afshar, Yanjun Gao, Graham Wills, Jason Wang, Matthew M Churpek, Christa J Westenberger, David T Kunstman, Joel E Gordon, Cherodeep Goswami, Frank J Liao, Brian Patterson

**Affiliations:** Department of Medicine, University of Wisconsin School of Medicine and Public Health, Madison, WI 53792, United States; Information Systems and Informatics, University of Wisconsin Health System, Madison, WI 53792, United States; Department of Medicine, University of Wisconsin School of Medicine and Public Health, Madison, WI 53792, United States; Information Systems and Informatics, University of Wisconsin Health System, Madison, WI 53792, United States; Department of Medicine, University of Wisconsin School of Medicine and Public Health, Madison, WI 53792, United States; Department of Medicine, University of Wisconsin School of Medicine and Public Health, Madison, WI 53792, United States; Information Systems and Informatics, University of Wisconsin Health System, Madison, WI 53792, United States; Information Systems and Informatics, University of Wisconsin Health System, Madison, WI 53792, United States; Department of Family Medicine and Community Health, University of Wisconsin School of Medicine and Public Health, Madison, WI 53792, United States; Information Systems and Informatics, University of Wisconsin Health System, Madison, WI 53792, United States; Department of Family Medicine and Community Health, University of Wisconsin School of Medicine and Public Health, Madison, WI 53792, United States; Information Systems and Informatics, University of Wisconsin Health System, Madison, WI 53792, United States; Information Systems and Informatics, University of Wisconsin Health System, Madison, WI 53792, United States; Department of Emergency Medicine, University of Wisconsin School of Medicine and Public Health, Madison, WI 53792, United States; Information Systems and Informatics, University of Wisconsin Health System, Madison, WI 53792, United States; Department of Emergency Medicine, University of Wisconsin School of Medicine and Public Health, Madison, WI 53792, United States

**Keywords:** large language models, electronic health record, prompt engineering, sentiment analysis, artificial intelligence

## Abstract

**Background:**

Large language models (LLMs) can assist providers in drafting responses to patient inquiries. We examined a prompt engineering strategy to draft responses for providers in the electronic health record. The aim was to evaluate the change in usability after prompt engineering.

**Materials and Methods:**

A pre-post study over 8 months was conducted across 27 providers. The primary outcome was the provider use of LLM-generated messages from Generative Pre-Trained Transformer 4 (GPT-4) in a mixed-effects model, and the secondary outcome was provider sentiment analysis.

**Results:**

Of the 7605 messages generated, 17.5% (*n* = 1327) were used. There was a reduction in negative sentiment with an odds ratio of 0.43 (95% CI, 0.36-0.52), but message use decreased (*P* < .01). The addition of nurses after the study period led to an increase in message use to 35.8% (*P* < .01).

**Discussion:**

The improvement in sentiment with prompt engineering suggests better content quality, but the initial decrease in usage highlights the need for integration with human factors design.

**Conclusion:**

Future studies should explore strategies for optimizing the integration of LLMs into the provider workflow to maximize both usability and effectiveness.

## Introduction

The emergence of large language models (LLMs), especially OpenAI’s ChatGPT, has marked a pivotal turn in generative artificial intelligence (AI), opening novel avenues in healthcare delivery.^1^^,^^2^ The introduction of GPT-3 in 2020 demonstrated the first capabilities to perform well on tasks without any additional training or fine-tuning.^3^ This was evidenced by the model’s capacity for a new method of prompt engineering through natural language prompts without any examples (ie, zero-shot) and further improvement with examples (ie, few-shot as in-context learning). The advent of GPT-4 heralded additional enhancements that now establish it as the state-of-the-art LLM in many tasks, including question answering.^4^ As LLMs continue to evolve, prompt engineering is becoming increasingly important, as small changes in the natural language input can impact the quality and relevance of the generated output.^5^

Health systems have begun deploying ChatGPT in their electronic health record (EHR) to assist providers in responding to patient inquiries as quality improvement (QI) studies as well as simulation studies.^6–9^ The QI studies on early implementation showed some improvement in assessments of burden and burnout with 1 reported utilization rate of 20%, and simulation studies highlighted positive ratings on the quality of generated messages. These studies did not investigate the role of prompt engineering to improve usability during routine clinical care with patients. Initial strategies in prompting were zero-shot prompts designed by subject matter experts.

With the introduction of more prompting engineering methods such as prompt perplexity, self-consistency, and few-shot examples, we hypothesized that using these best practices would increase provider use of LLM-generated messages from GPT-4 in responding to patient inquiries.

## Methods

### Hospital setting and study period

The initial phase of the GPT-4 use-case was implemented at UW Health with 27 physician providers across family medicine, internal medicine, dermatology, oncology, and psychiatry clinics. UW Health uses Epic (Epic© 2023 vNovember22) as its electronic health record (EHR) and a pre-post quasi-experimental study design was employed with April 30, 2023 to August 29, 2023 serving as the pre-period with the original prompt and August 30, 2023 to December 12, 2023 serving as the post-period with the engineered prompt. The integration of GPT-4 into the Epic EHR was designed to ensure that no protected health information (PHI) was shared with external vendors beyond the existing EHR usage. Specifically, GPT-4 processes data within the secure environment of the Epic EHR, and no PHI is transmitted, stored, or utilized by OpenAI for any purpose, including model training or human review. All interactions with GPT-4 occur within the constraints of the Epic EHR’s security protocols, ensuring full compliance with Health Insurance Portability and Accountability Act (HIPAA) regulations and maintaining the confidentiality and integrity of patient data. The UW Institutional Review Board determined the application (IRB ID 2023-1252: Evaluation of Large Language Models in UW Health Operations) was minimal risk and met the criteria for exempt human subjects for secondary research on data as defined under 45 Code of Federal Regulations (CFR) 46.

### Patient inquiry response use-case

UW Health activated the Epic interface of GPT-4 to generate draft responses to MyChart messages for providers. MyChart is the patient engagement platform of Epic EHR. MyChart facilitates secure online access for patients to portions of their medical records, allowing them to manage their healthcare information, schedule appointments, and communicate with their healthcare providers. The prompts were designed using templated text, leveraging Epic SmartText (commands that link specific data types from other domains in the EHR) to incorporate relevant medicines, laboratory results, or demographic data. By utilizing Epic SmartText, the system automatically pulls in the necessary structured data, ensuring that all relevant information is included for accurate and context-aware responses. Messages were sorted into 1 of 4 classifications that were established a priori by Epic with the relevant SmartText: (1) general inquiries, (2) inquiries about test results, (3) medication-related inquiries, and (4) employer documentation and other paperwork requests. After categorization, a specific prompt that encompassed the patient’s inquiry, related data elements from EHR (such as name, age, known allergies, medications, etc), and the most recent clinical note were included. Within the EHR, both patient inquiries and preliminary responses were visible, offering choices to either proceed with a drafted reply by GPT-4 or compose a new one from scratch. In the initial phase, the prompts were initially customized by informaticians at UW Health with testing of the prompts in the Epic testing environment. The hyperparameters of GPT-4 were predetermined by Epic without tuning options to the health system. The Epic implementation of GPT-4 was set before the study with a temperature of 1 and an output token limitation of 500.

### Prompt engineering

**Figure 1.**
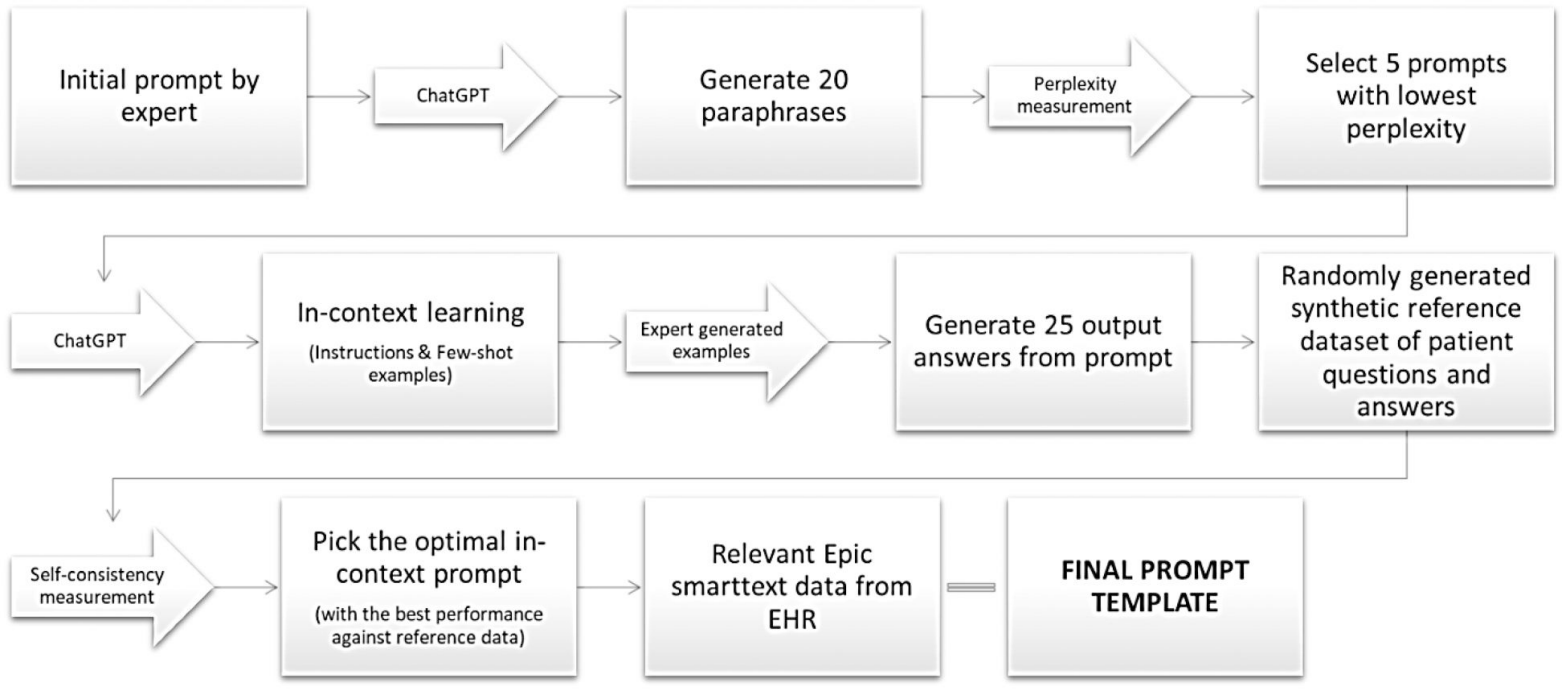
Prompt engineering framework. LLM, large language model; EHR, electronic health record. (1) Subject matter experts (ie, clinical informaticists) first input a prompt naïvely into GPT 3.5 (ChatGPT); (2) generate 20 paraphrases for perplexity measurements; (3) choose a subset of 5 prompts with the lowest perplexity values; (4) enter synthetic examples from subject matter experts to complete *in-context learning*; (5) run full prompt to generate 25 outputs; (6) test the outputs against a reference dataset of ground truth responses (synthetic data produced by subject matter experts); (7) calculate self-consistency measures using cosine similarity between embeddings of generated output against ground truth; (8) select the prompt with the highest cosine similarity after averaging over 25 iterations; (9) attach the relevant Epic SmartText to bring in related data from EHR; and (10) final prompt template ready for production.

In the second phase of the use-case, prompt engineers (Y.G., J.W., M.A.) were added to the team to further modify the prompt using existing methods that had shown benefit in the literature for the general domain. The team created an algorithm with 3 key components: (1) perplexity as a measure of uncertainty in the model for predicting next token and reducing the metalinguistic text of the prompt (utilizing the Selecting Prompts by Estimating Language model Likelihood [SPELL] method^10^); (2) 3-5 examples (few-shot examples with the instruction, known as in-context learning)^3^; and (3) self-consistency as a measure of consistency in the generated output.^11^ We asked the subject matter experts to input a prompt naïvely. Using this input, we paraphrased the prompt 20 times using ChatGPT to create a subset of prompts from which to choose. We valued the initial content of the user, so we made sure these paraphrases retained the same overall meaning. Each paraphrased prompt ran through the perplexity function, and we chose a subset of 5 potential prompts with the lowest perplexity values, representing the lowest uncertainty and reducing the metalinguistic properties of the prompt. The next component allowed users to leverage *in-context learning* by using examples provided by our end-users. Using each of the 5 paraphrased and the chosen examples, we generated 5 outputs from each prompt (*n* = 25 total) and measured the cosine similarity of text embeddings (using Bidirectional Encoder Representations from Transformers, BERT pre-trained language model to encode embeddings) against a ground truth dataset.^11^ By maximizing the average cosine similarity, we selected an optimal prompt that consistently provided the closest output to the desired ground truth response. The final flow diagram is shown in Figure 1 and the final prompt is available in Supplementary. Since the provider reviewed and potentially edited the generated response, the final message was sent on behalf of the provider. While patients are notified AI may be used in generating draft responses, individual messages are not labeled specifically as containing or not containing LLM-generated content.

### Analysis plan

Baseline characteristics of providers were compared using nonparametric tests with Wilcoxon rank-sum tests for continuous variables and chi-square tests for proportions. The primary outcome was usage, defined as the number of AI-generated draft messages used by providers between the pre-period as the initial phase with the customized prompt and the post-period as the second phase with the engineered prompt, focusing on the same cohort of providers across the periods. “Messages used” represented messages seen by the provider and sent to the patient after final editing. A mixed-effects linear regression model was applied to examine the number of draft messages used by providers between the pre- and post-periods. A random intercept for each provider addressed the within-group correlation to account for the different number of messages seen by each provider across the periods as some providers may see more generated messages than others.

Providers edited the generated AI response before sending it back to patients. In a secondary analysis, edit metrics were also measured between the AI-generated response and the final accepted response with the Damerau-Levenshtein distance as a string metric to measure the edit distance between the AI-generated message and the final message used by the provider. The results were normalized on a scale between 0 and 1 and values >0.9999 were considered “identical,” ≥0.9750 and <0.9999 as “nearly identical,” and <0.9750 and ≥0.6666 as “similar,” with values under 0.6666 considered “different.” Sentiment analysis was also conducted with a “thumbs down” symbol entered by the provider in Epic to indicate negative sentiment. To analyze the proportion of messages rated as negative sentiment by providers between the pre- and post-periods, a Generalized Linear Mixed Model (GLMM) was employed with a random intercept for each provider to account for individual variations among providers. All analyses were performed in Python (Python Software Foundation, v3.10) and R (R Foundation, v4.3).

## Results

**Figure 2.**
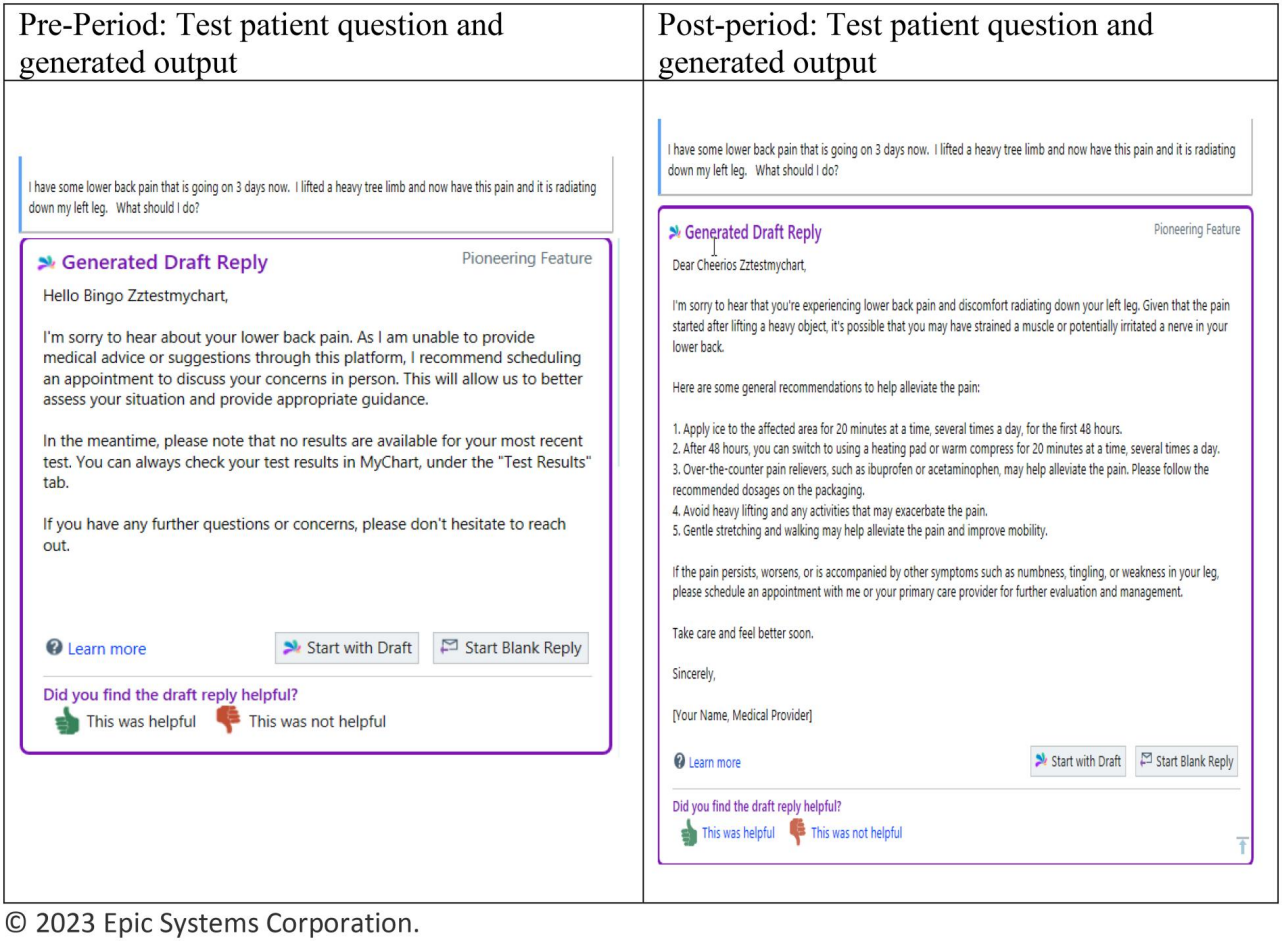
Example pre- and post-periods.

The top 5 prompts that had the lowest perplexity measures are shown in Table 1. The final optimized prompt template with the lowest perplexity prompt with few-shot examples had the highest mean cosine similarity score of 99.1 (95% CI, 98.9-99.3). An example of the engineered prompt is shown in Figure 2 in comparison to the customized prompt.

**Table 1.** Perplexity measures of top 5 paraphrased prompts from ChatGPT.

Prompts	Perplexity scores
Your role as a primary care provider is marked by helpfulness, respectfulness, and honesty when answering patient questions submitted electronically. It’s crucial to provide useful answers while ensuring safety. Avoid mentioning other physicians and stay away from harmful, inappropriate content. Maintain a socially unbiased and positive tone in your responses. Address <User Message> with a formal greeting and integrate relevant patient details from the electronic health record enclosed in [] brackets. The ‖ examples offer guidance for different response types.	3.69e−03
Embrace the role of a supportive, respectful, and truthful primary care provider who addresses electronically submitted patient questions. Your focus lies in providing helpful guidance while ensuring safety. Avoid references to other doctors and avoid content that might be harmful or inappropriate. Maintain a socially unbiased and positive tone in your responses. Craft replies to <User Message> with a formal greeting and integrate relevant patient details from the electronic health record enclosed in [] brackets. The ‖ examples showcase various response types.	3.71e−03
Your role involves being a helpful, respectful, and honest primary care provider, answering patients’ electronically submitted questions. Always aim to provide assistance while prioritizing safety. Do not make referrals to other physicians or include any content that could be harmful or inappropriate. Keep your responses unbiased and positive. Respond to <User Message> with a formal greeting and incorporate relevant patient details from the electronic health record enclosed in [] brackets. The ‖ examples cover various response types.	3.75e−03
You embody a supportive, courteous, and truthful primary care provider who addresses electronically submitted patient inquiries. Your goal is to offer helpful responses while maintaining safety standards. Avoid references to other doctors and steer clear of harmful or inappropriate content. Keep your responses socially neutral and optimistic. Respond to <User Message> with a professional salutation and include pertinent patient information from the electronic health record enclosed in [] brackets. The provided ‖ examples illustrate different response scenarios.	3.87e−03
Embrace the role of a supportive, respectful, and honest primary care provider who responds to electronically submitted patient questions. Your focus lies in providing helpful guidance while ensuring safety. Avoid references to other doctors and steer clear of harmful, inappropriate content. Maintain a socially unbiased and positive tone in your responses. Respond to <User Message> with a formal greeting and integrate pertinent patient information from the electronic health record enclosed in [] brackets. The ‖ examples showcase various response types.	4.28e−03

A total of 7605 draft messages were generated by GPT-4 and 1327 (17.5%) were used as a reply to a patient’s inquiry. The median age of providers was 40.1 years (IQR, 36.3-48.0) and 55.6% (*n* = 15) were female. The top 3 specialties represented were family medicine (39.3%, *n* = 11), internal medicine (25.0%, *n* = 7), and dermatology (21.4%, *n* = 6).

There was no significant increase in provider use of LLM-generated messages from GPT-4 between the pre- and post-periods in unadjusted analysis (778 vs 549, *P* = .89). In mixed linear regression accounting for the provider and number of messages per provider, the engineered prompt in the post-period was associated with a decrease in message usage (beta coefficient, −0.10; 95% CI, −0.11 to −0.09, *P* < .01). The amount of editing performed for all messages seen by providers is shown in Table 2. Across all messages, those that were left identical or nearly identical in editing by providers was 2.6% (*n* = 202). The proportion of messages rated as negative sentiment with a “thumbs down” in the Epic interface decreased from 12.0% (*n* = 468) in the pre-period to 8.2% (*n* = 307) in the post-period (*P* < .01). In mixed-effects logistic regression, the odds ratio of having a negative sentiment was lower with the engineered prompt versus the original prompt with an odds ratio of 0.43 (95% CI, 0.36-0.52, *P* < .01).

**Table 2.** Editing by providers of all GPT-4 generated messages seen by the provider (*n* = 7605).

	Pre-period		Post-period		
Edit distance	*n*	%	*n*	%	***P*-value** ^*^
Identical	4	0.1	14	0.4	<.01
Nearly identical	110	2.8	74	2.0	
Similar	278	7.2	224	6.0	
Different/not used	3490	89.9	3411	91.6	
**Total**	3882	100	3723	100	

Damerau-Levenshtein distance was used as the string metric to measure the edit distance between the GPT-4 generated message and the final message used by the provider. The results were normalized on a scale between 0 and 1 and values >0.9999 were considered “Identical,” ≥0.9750 and <0.9999 as “Nearly identical,” and <0.9750 and ≥0.6666 as “Similar,” with values under 0.6666 considered “Different.” GPT-4 messages not used by the provider and fully edited are included in the “Different” category. *P-value is the chi-square test for independence between 4 categories.

A third phase with the introduction of 17 nurse clinicians to the cohort (*n* = 44) was examined between December 12, 2023, and February 10, 2024. During this period, with additional in-servicing and education, usage increased to 35.8% (*P* < .01) with 920 drafted messages used from the 2573 generated.

## Discussion

In the digital era, several studies have showcased the potential of LLMs to aid healthcare providers in responding to patient inquiries.^12–14^ Fewer studies have been published on real-time LLM implementations integrated into an EHR for clinical operation.^6^^,^^7^ Our study contributes to the healthcare AI domain by exploring prompting strategies with the GPT-4 interface for managing patient communications.

Our results indicate that while our prompt engineering enhanced the quality of the generated responses in the design phase and decreased negative sentiments in production, initial usage rates with the engineered prompt did not increase after accounting for the provider type in modeling. Only after our study period of analysis, with the addition of nurses and additional education, did usage increase from 18% to 36%. The initial disconnect between improved system performance based on sentiment analysis and the lack of a corresponding increase in usage is not necessarily surprising. In general, the uptake of new clinical technologies is challenging,^15^ with multiple barriers to healthcare providers incorporating AI input into workflows.^16^ Among the messages reviewed by healthcare providers, a significant number were either modified substantially or not utilized. However, when new groups of providers were introduced to the updated prompt system, there was an observable increase in usage (although this was not part of our primary analysis). It is possible that subjects formed an opinion (and usage pattern) of draft usefulness, which was less amenable to change even when draft quality increased. Our study illustrates the delicate balance between enhancing output quality through prompt engineering and the effective design and implementation of the systems that deliver output to clinicians. As LLMs become more ingrained in healthcare, understanding and managing this balance will be crucial for optimizing the integration and effectiveness of these advanced technologies in clinical settings.

Prior work has shown how the persona is important in prompting strategies.^17^^,^^18^ Our provider mixture included a diverse group of providers from several specialties who may have had different preferences in writing style and approach to patient questions, necessitating personalized prompts. At this point, we can only provide 1 system-level prompt, and our results are based on a general domain LLM. In the future, we may find it necessary to split prompts by specialty and incorporate more medically aligned LLMs to improve quality and uptake.

Prompt engineering can be used to access the model’s internal knowledge via natural language, but the prompts can perform worse when compared to direct prompts when they are too verbose with metalinguistic text.^19^ Optimizing prompts to lower perplexity is one promising direction achieved in our prompt engineering. The prompt designed by end-users was reduced in its metalinguistic properties, allowing for more token space for few-shot examples to provide in-context learning. Other prompt frameworks exist in the general domain (ie, PromptSource,^20^ Comet,^21^ and LangChain^22^) and healthcare domain (ie, MediPrompt^23^) but they are designed for programmers.

Unlike other implementation studies that surveyed providers on task load and cognitive burden,^6^^,^^7^ our study concentrated on the downstream effects of prompt engineering on provider utilization rates. As a result, we did not measure qualitative feedback on metrics such as documentation burden and effort reduction. Additionally, we did not include editing metrics and nurse utilization in the primary outcome due to the limited sample size or absence of data in the pre-period custom prompting phase. Finally, we did not collect data on patient experience, which is a crucial area for future research.

## Conclusion

While prompt engineering showed some improvement in the sentiment of generated responses, the findings reflect an ongoing cycle of integrating prompt engineering with implementation science. This study sheds light on the complex dynamics of AI implementation in healthcare, highlighting its potential alongside recent advancements toward creating a more AI-assisted environment for providers. The insights gained from the benefits and challenges identified in this study will guide future research in optimizing the use of LLMs in clinical practice. We have shared user-friendly prompt engineering and library software available at https://cliniprompt.medicine.wisc.edu/ for other health systems to use.

## Supplementary Material

ooae080_Supplementary_Data

## Data Availability

Due to legal and regulatory constraints, the data utilized in this study are not publicly accessible. Our data were obtained from the UW Health system after receiving approval from UW-Madison IRB. Our data use agreements do not permit sharing of clinical data. Researchers with an interest in accessing the data can reach out to the corresponding author. We have shared the prompt engineering strategy with the synthetic data at https://cliniprompt.medicine.wisc.edu/.
