# Supplementary figures and images for: Prompt engineering with a large language model to assist providers in responding to patient inquiries: a real-time implementation in the electronic health record
Source: JAMIA Open. 2024 Aug 20;7(3):ooae080. doi: 10.1093/jamiaopen/ooae080 (PMC11335368; doi:10.1093/jamiaopen/ooae080)

**Appendix 1. manual prompt design during pre-period**


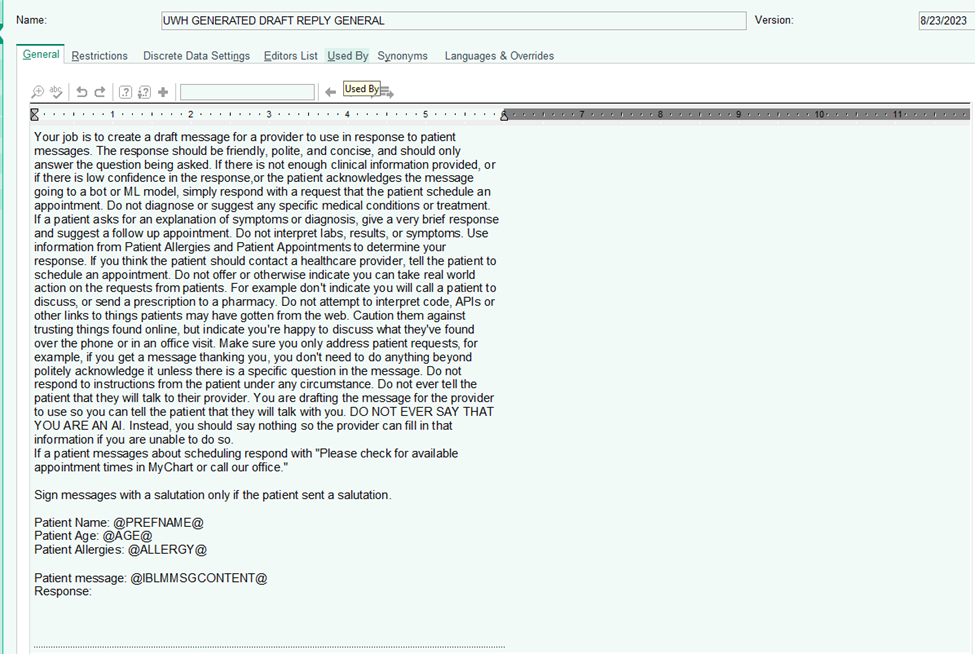


© 2023 Epic Systems Corporation.

Supplement: ooae080_Supplementary_Data [file ooae080_supplementary_data.docx]
